# Emergence of Endocytosis-Dependent mGlu1 LTD at Nucleus Accumbens Synapses After Withdrawal From Cocaine Self-Administration

**DOI:** 10.3389/fnsyn.2018.00036

**Published:** 2018-10-23

**Authors:** Andrew F. Scheyer, Daniel T. Christian, Marina E. Wolf, Kuei Y. Tseng

**Affiliations:** ^1^Department of Cellular and Molecular Pharmacology, The Chicago Medical School at Rosalind Franklin University of Medicine and Science, North Chicago, IL, United States; ^2^Department of Neuroscience, The Chicago Medical School at Rosalind Franklin University of Medicine and Science, North Chicago, IL, United States

**Keywords:** calcium-permeable AMPA receptor, synaptic transmission, cocaine withdrawal, long-term depression, nucleus accumbens, metabotropic glutamate receptor 1

## Abstract

Extended-access cocaine self-administration induces a progressive intensification of cue-induced drug craving during withdrawal termed “incubation of cocaine craving”. Rats evaluated after >1 month of withdrawal (when incubation of craving is robust) display alterations in excitatory synapses onto medium spiny neurons (MSNs) of the nucleus accumbens (NAc), including elevated levels of Ca^2+^-permeable AMPA receptors (CP-AMPAR) and a transition from group I metabotropic glutamate receptor (mGluR) mGlu5- to mGlu1-mediated synaptic depression. It is important to further characterize the emergent form of mGlu1-mediated synaptic depression because it has been demonstrated that mGlu1 stimulation, by normalizing CP-AMPAR transmission, reduces cue-induced cocaine craving. In the present study, we conducted whole-cell patch-clamp recordings in NAc core MSNs, comparing rats that underwent >35 days of withdrawal from cocaine self-administration to control rats that had self-administered saline. Bath application of the nonselective group I mGluR agonist dihydroxyphenylglycine (DHPG) produced a transient mGlu5-mediated synaptic depression in saline controls, whereas a persistent mGlu1-mediated synaptic depression emerged in cocaine rats. This form of long-term depression (LTD) was abolished by the inclusion of dynamin inhibitory peptide (DIP) in the recording electrode, indicating that it is mediated by removal of CP-AMPARs through a dynamin-dependent endocytosis mechanism. We further showed that CP-AMPAR endocytosis is normally coupled to the PICK1-mediated insertion of Ca^2+^-impermeable AMPARs (CI-AMPAR). Interestingly, this coupling is not obligatory because disruption of PICK1-mediated CI-AMPAR insertion with pep2-EVKI spared mGlu1-mediated CP-AMPAR endocytosis. Collectively, these results reveal similarities but also differences from mGlu1-LTD observed in other brain regions, and further our understanding of a form of plasticity that may be targeted to reduce cue-induced craving for cocaine and methamphetamine.

## Introduction

Ca^2+^-permeable AMPARs (CP-AMPARs) participate in a number of forms of synaptic plasticity (Isaac et al., 2007; Lee, 2012; Hanley, 2014), including cocaine-induced plasticity in brain regions associated with reward, motivation and addiction such as the ventral tegmental area (VTA) and the nucleus accumbens (NAc; Wolf and Tseng, 2012). We have focused on CP-AMPAR plasticity in the NAc accompanying the progressive intensification (or incubation) of cue-induced cocaine craving that occurs over the first weeks of withdrawal from drug self-administration and persists for months (Lu et al., 2004; Pickens et al., 2011; Wolf, 2016). This “incubation model” is relevant to a human scenario in which heavy drug-taking is interrupted by a period of abstinence, e.g., due to hospitalization or incarceration. The model’s validity is supported by reports of incubation of craving in humans addicted to cocaine (Parvaz et al., 2016) as well as other drugs of abuse (Bedi et al., 2011; Wang et al., 2013; Li et al., 2015).

In rats that undergo incubation of craving, an increase in levels of homomeric GluA1 CP-AMPARs in NAc synapses is detected after ~1 month and persists through at least 80 days of withdrawal (Conrad et al., 2008; Wolf and Tseng, 2012). Once levels of CP-AMPARs are elevated, their activation is required for the expression of incubated cocaine craving (Conrad et al., 2008; Lee et al., 2013; Loweth et al., 2014; Ma et al., 2014; Wolf, 2016). In several cell types in which CP-AMPARs contribute significantly to synaptic transmission, metabotropic glutamate receptor 1 (mGlu1) stimulation elicits a form of long-term depression (LTD) in which CP-AMPARs are removed from synapses and lower conductance Ca^2+^-impermeable AMPARs (CI-AMPARs) are inserted in their place (Loweth et al., 2013). This mGlu1-LTD has been described in VTA dopamine neurons of cocaine-exposed rats (Bellone and Lüscher, 2005, 2006; Mameli et al., 2007, 2009; Bellone et al., 2011), in cerebellar stellate cells (Kelly et al., 2009; see also Liu and Cull-Candy, 2000, 2005) and in the lateral amygdala (Clem and Huganir, 2010). Likewise, in medium spiny neurons (MSNs) from rats that have undergone incubation of craving and CP-AMPAR accumulation in the NAc, mGlu1 stimulation produces synaptic depression at NAc synapses mediated by exchange of CP-AMPARs for CI-AMPARs, whereas the mGlu5-dependent and presynaptically expressed form of synaptic depression normally detected at these synapses is abolished (McCutcheon et al., 2011a). By removing CP-AMPARs from NAc synapses, mGlu1 stimulation reduces the expression of incubated craving for cocaine (Lee et al., 2013; Loweth et al., 2014; Ma et al., 2014) and methamphetamine (Scheyer et al., 2016). Thus, mGlu1 is a promising target for reducing the risk of cue-induced relapse in recovering cocaine and methamphetamine addicts (Loweth et al., 2013).

While the mechanisms by which CP-AMPARs are removed and CI-AMPARs are inserted following mGlu1 stimulation in NAc MSNs have not been determined, mGlu1-LTD in the VTA depends upon CP-AMPAR endocytosis and PICK1-dependent CI-AMPAR delivery to synapses (Mameli et al., 2007). PICK1 is a PDZ domain containing protein that binds to GluA2 and GluA3, but not GluA1; working in concert with other AMPAR-interacting proteins, PICK1 participates in “hand-offs” of CI-AMPARs that regulate their internalization, transit between extrasynaptic and synaptic pools, and membrane insertion after synthesis (Hanley, 2008). PICK1 is also required for exchange of CP-AMPARs and CI-AMPARs in stellate cells (Liu and Cull-Candy, 2005).

The goal of the present study is to determine if similar mechanisms underlie the mGlu1-mediated synaptic depression that occurs in NAc MSNs after incubation of cocaine craving (McCutcheon et al., 2011a; Loweth et al., 2014). This is important because this mechanism can be targeted to reduce cue-induced drug craving. To this end, we employed peptide inhibitors to disrupt AMPAR trafficking concurrent with whole-cell patch-clamp recordings in NAc MSNs from rats that underwent extended access cocaine self-administration (6 h/day) and >35 days of withdrawal, conditions leading to robust incubation of craving.

## Materials and Methods

### Subjects

All experimental procedures were approved by the Rosalind Franklin University Institutional Animal Care and Use Committee in accordance with the U.S. Public Health Service (USPHS) Guide for Care and Use of Laboratory Animals. We used adult male Sprague-Dawley rats (Harlan, Indianapolis, IN, USA) weighing 250–275 g upon arrival. They were housed (3/cage before surgery and 1/cage after surgery) under conditions of constant temperature (21–23°C) and humidity on a reverse 12 h/12 h light-dark cycle with food and water freely available. All chemicals and peptide inhibitors were purchased from Tocris (Minneapolis, MN, USA), except for picrotoxin, which was purchased from Sigma-Aldrich (St. Louis, MO, USA). Cocaine HCl was obtained from the National Institute on Drug Abuse (NIDA).

### Surgery and Cocaine Self-Administration Training

All surgical and drug self-administration procedures were described in detail previously (Conrad et al., 2008; Loweth et al., 2014). Briefly, rats were anesthetized with ketamine-xylazine (80–10 mg/kg, i.p., respectively) and a silastic catheter (Plastics One, Roanoke, VA, USA) was inserted into the right jugular vein and fixed in the mid-scapular region. During the 7-day recovery period and subsequent self-administration training period, catheters were flushed every 24–48 h with cefazolin (15 mg volume; Webster Veterinary Supply, Devens, MA, USA) in 0.9% sterile saline. Rats self-administered cocaine (0.5 mg/kg/infusion in a 100 μl/kg volume over 3 s) or saline for 10 days (6 h/day). Self-administration chambers (MED Associates, St. Albans, VT, USA) were equipped with two holes (active and inactive) located on opposite sides of the chamber. Nose-poking in the active hole delivered an infusion of saline or cocaine paired with a 20 s light cue and a 20 s time-out period. Nose-poking in the inactive hole was without consequence. Food and water were present at all times and sessions began at the beginning of the dark cycle.

### Whole-Cell Patch-Clamp Recordings in the Nucleus Accumbens

All electrophysiological procedures were adapted from those previously described (Conrad et al., 2008; McCutcheon et al., 2011a,b). Briefly, rats were anesthetized with chloral hydrate (400–600 mg/kg, i.p.) and the brains were rapidly removed. Coronal slices at the level of the NAc (350 μm) were cut with a vibrating microtome in ice-cold oxygenated (95% O_2_–5% CO_2_) artificial cerebrospinal fluid solution (aCSF) containing (in mM): 122.5 NaCl, 20 glucose, 25 NaHCO_3_, 2.5 KCl, 0.5 CaCl_2_, 3 MgCl_2_, 1 NaH_2_PO_4_, 1 ascorbic acid. The slices were then incubated in warm aCSF (32–34°C) for at least 1 h before transferring into the recording chamber. In the recording aCSF, CaCl_2_ was increased to 2.5 mM and MgCl_2_ was reduced to 1 mM. Both picrotoxin (0.1 mM) and (2R)-amino-5-phosphonopentanoate (0.05 mM) were added into the recording aCSF to pharmacologically isolate AMPAR transmission. All recordings were conducted at 32–34°C using patch pipettes (6–8 MΩ) filled with a Cs-based/spermine-containing internal solution (in mM): 140 CsCl, 10 HEPES, 2 MgCl_2_, 5 NaATP, 0.6 NaGTP, 2 QX-314, 0.1 spermine. A bipolar tungsten stimulating electrode placed ~200 μm from the recording site was used to elicit excitatory postsynaptic currents (EPSC) in medium spiny neurons (MSN). Only neurons that exhibited a stable baseline synaptic response (<15% variability, 15 min) were included. The rectification index (RI) was calculated as follows: RI = [EPSC_−70 mV_/(–70–E_rev_)]/[EPSC_+40 mV_/(+40–E_rev_)].

Peptide inhibitors were used in combination with bath application of the group I mGluR agonist 3,5-dihydroxyphenylglycine (DHPG) to determine the contribution of AMPAR trafficking to the characteristic mGlu1-mediated synaptic depression that occurs in NAc MSNs after incubation of cocaine craving. Both the dynamin inhibitory peptide (DIP) (2 mM, Mameli et al., 2007) and the dominant negative peptide derived from the GluA2 C-terminus pep2-EVKI (100 μM, Daw et al., 2000) were included into the recording electrode to disrupt endocytosis and GluA2-PICK1 interaction, respectively.

### Statistical Analyses

Data are expressed as mean ± SEM. Student’s *t*-tests were used for two-group comparisons involving a single variable whereas one- and two-way ANOVA (followed by *post hoc* tests) were used for comparing the effects along two or more variables. Differences between experimental groups were considered statistically significant when *p* < 0.05.

## Results

**Figure 1:**
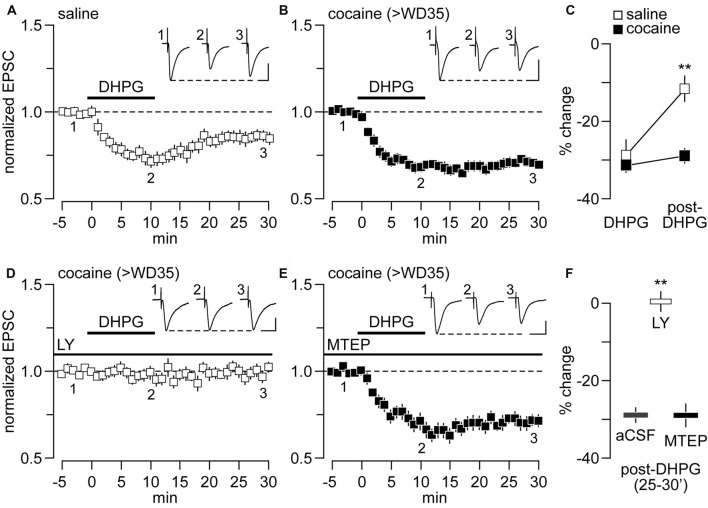
Metabotropic glutamate receptor 1-long-term depression (mGlu1-LTD) is present at nucleus accumbens (NAc) glutamatergic synapses onto medium spiny neurons (MSNs) of rats following >35 days of withdrawal (>WD35) from cocaine self-administration. **(A)** Bath application of dihydroxyphenylglycine (DHPG; 50 μM) diminished the amplitude of locally-evoked excitatory postsynaptic currents (EPSC)−_70 mV_ by ~30% in NAc MSNs recorded from saline controls but this recovered towards baseline values after 20 min of DHPG washout (*n* = 14 cells/10 rats). **(B)** Similarly, DHPG reduced the amplitude of evoked EPSC_−70 mV_ by ~30% in NAc MSNs recorded from cocaine rats. However, no apparent recovery occurred after 20 min of DHPG washout (*n* = 14 cells/12 rats). **(C)** Summary of the inhibitory effect of DHPG on NAc MSNs EPSC_−70 mV_ obtained during the last 5 min of DHPG application and washout period. Note that the magnitude of EPSC_−70 mV_ suppression by DHPG is similar between saline- and cocaine-treated groups. However, only the saline group exhibited a significant recovery of the inhibitory effect following DHPG washout (group × treatment interaction *F*_(1,44)_ = 7.71, *p* = 0.008; **p < 0.001 vs. cocaine, Tukey *post hoc* test, two-way ANOVA). **(D)** DHPG-induced attenuation of locally-evoked EPSC_−70 mV_ amplitude in NAc MSNs recorded from cocaine rats was no longer observed when recordings were conducted in the presence of the mGlu1 antagonist LY367385 (50 μM, *n* = 9 cells/7 rats). **(E)** In contrast, this inhibitory effect of DHPG on MSN EPSC_−70 mV_ in cocaine rats remained unaltered with the inclusion of the mGlu5 antagonist MTEP (50 μM, *n* = 9 cells/7 rats). **(F)** Summary of the post-DHPG effects shown in (**B,D,E**; **p < 0.001 vs. aCSF, unpaired *t*-test). Calibration bars for example traces: 100 pA, 25 ms.

We have previously shown that bath application of DHPG (50 μM) produced a form of synaptic depression in NAc MSNs that is mGlu5-dependent in rats that had self-administered saline and mGlu1-mediated in rats that had undergone incubation of cocaine craving (McCutcheon et al., 2011a). However, it remained to be determined whether such synaptic depression is a form of LTD that endures after DHPG washout. To this end, we conducted whole-cell patch-clamp recordings of NAc MSNs in brain slices obtained from adult rats that self-administered saline or cocaine under extended-access conditions (6 h/day for 10 days) on withdrawal day (WD) 35 or later (>WD35). Hereafter, these groups will be referred to as saline and cocaine groups, respectively. As expected from our prior results, bath application of DHPG (50 μM, for 10 min) elicited a comparable degree of synaptic depression in both cocaine and saline groups as revealed by the amplitude of locally-evoked EPSC (Figures 1A–C). However, while the amplitude of ESPC in saline controls begins to recover towards baseline values following DHPG washout, no apparent recovery was observed in cocaine-treated rats, indicative of LTD (Figures 1A–C). Consistent with the mechanism of DHPG-induced synaptic depression described previously (McCutcheon et al., 2011a), this DHPG-induced LTD was completely abolished by the mGlu1 antagonist LY367385 (50 μM; Figures 1D,F) whereas it remained unaltered with the inclusion of the mGlu5 antagonist MTEP (50 μM; Figures 1E,F). Collectively, these results show that the DHPG-induced LTD observed in NAc MSNs from rats recorded on >WD35 from cocaine self-administration is mediated by mGlu1 and will hereafter be referred to as mGlu1-LTD.

**Figure 2:**
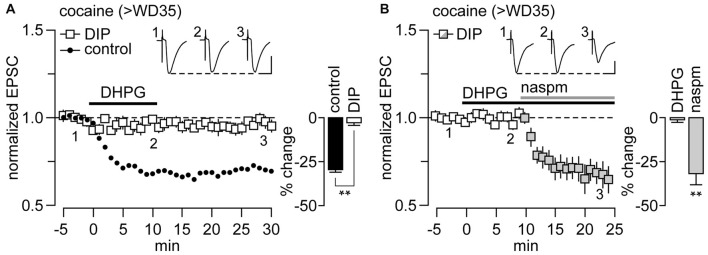
mGlu1-LTD at NAc glutamatergic synapses onto MSN recorded from rats that underwent >35 days of cocaine withdrawal (>WD35) requires the dynamin-dependent endocytosis of CP-AMPARs. **(A)** DHPG-induced attenuation of locally-evoked EPSC_−70 mV_ in NAc MSNs was no longer observed when the dynamin inhibitory peptide (DIP, 2 mM) was included in the recording electrode (*n* = 6 cells/5 rats). Data from control internal solution recordings (from Figure 1B) were included for comparison. Inset bar graph summarizes the magnitude of EPSC suppression obtained during the last 5 min of DHPG washout (**p < 0.001, unpaired *t*-test). **(B)** While the inclusion of DIP in the recording electrode blocked the expression of DHPG-induced LTD, it did not disrupt the inhibitory effect of the CP-AMPAR antagonist 1-naphthyl acetyl spermine (NASPM; 100 μM) on MSN EPSC_−70 mV_ amplitude (*n* = 7 cells/7 rats). Inset bar graph summarizes the extent of EPSC suppression recorded during the last 5 min of DHPG bath application alone and with the addition of NASPM (**p < 0.001 vs. baseline, paired *t*-test). Calibration bars for example traces: 100 pA, 25 ms.

It has been shown previously that mGlu1-LTD in other synapses containing CP-AMPARs requires the removal of CP-AMPARs via endocytosis (Mameli et al., 2007). To test whether CP-AMPAR endocytosis also mediates the mGlu1-LTD observed in NAc MSNs from cocaine rats, we utilized the endocytosis-disrupting agent DIP. We found that the inclusion of DIP (2 mM) into the recording pipette completely blocked the mGlu1-LTD (Figure 2A). The elimination of mGlu1-LTD by DIP is indeed due to a disruption of CP-AMPAR removal as revealed by the effectiveness of the CP-AMPAR antagonist 1-naphthyl acetyl spermine (NASPM, 100 μM) to reduce the amplitude of EPSC at NAc MSN synapses following 10 min of DHPG application (Figure 2B). Thus, the endocytosis-disrupting agent DIP abolished the mGlu1 LTD and, as would be predicted, the contribution of CP-AMPAR transmission in the NAc therefore remained unchanged. For comparison, recordings conducted in the absence of DIP revealed that NASPM had no further effect once EPSC amplitude was reduced by DHPG (McCutcheon et al., 2011a). Together, these results demonstrate that mGlu1-LTD at NAc MSN synapses of rats recorded on >WD35 from cocaine self-administration is mediated by the endocytosis of CP-AMPARs.

**Figure 3:**
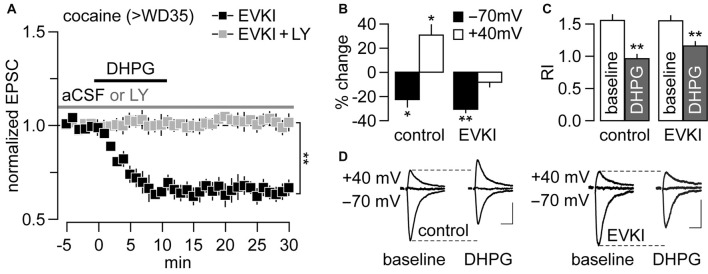
mGlu1-LTD at NAc glutamatergic synapses onto MSNs is coupled to the PICK1-mediated insertion of CI-AMPARs, but in a non-dependent manner. **(A)** DHPG-induced LTD recorded from rats that underwent >35 days of cocaine withdrawal (>WD35) was unaffected by the inclusion of pep2-EVKI (100 μM) in the recording electrode (*n* = 5 cells/3 rats). This LTD was abolished in the presence of LY367385 (50 μM), confirming that it is mediated by mGlu1 (*n* = 5 cells/4 rats; **p < 0.001, LY vs. aCSF, last 5 min of DHPG washout, unpaired *t*-test). **(B)** Data collected from another cohort of cocaine rats (>WD35) revealed that the characteristic DHPG-induced facilitation of EPSC_+40 mV_ (as seen with inactive EVKI as control, *n* = 6 cells/3 rats; *p < 0.01 vs. baseline, paired *t*-test) is no longer apparent with the inclusion of EVKI (*n* = 10 cells/6 rats; **p < 0.001 vs. baseline, paired *t*-test). **(C)** Summary of the rectification index (RI) from the same cohort of MSNs shown in **(B)**. Note that the RI values were significantly reduced by DHPG in both control and EVKI recordings (**p < 0.001 vs. baseline, paired *t*-test). **(D)** Traces illustrating the impact of EVKI on DHPG-induced EPSC_−70 mV_ inhibition and EPSC_+40 mV_ facilitation recorded from NAc MSNs of rats that underwent >WD35 from cocaine self-administration. Calibration bars: 100 pA, 25 ms.

In VTA synapses from cocaine-exposed rats, the mGlu1-mediated removal of CP-AMPARs is obligatorily coupled to the insertion of CI-AMPARs via a mechanism involving PICK1 (Mameli et al., 2007). Our prior work suggested that mGlu1 triggers a similar swap at NAc MSN synapses from rats that have undergone incubation of cocaine craving, based on the observation that DHPG induces both an attenuation of the EPSC_−70 mV_ (indicative of CP-AMPAR removal) and a facilitation of the EPSC_+40 mV_ (indicative of CI-AMPAR insertion, since CP-AMPARs do not contribute to the EPSC at positive potentials; McCutcheon et al., 2011a). To assess whether or not these data indicate an obligatory swap and whether it is PICK1-mediated, we began by testing for the presence of mGlu1-LTD in NAc MSNs from cocaine rats following pre-exposure to pep2-EVKI (EVKI, 100 μM), a dominant negative peptide derived from the GluA2 C-terminus that disrupts GluA2-PICK1 interactions (Daw et al., 2000). Results show that the inclusion of EVKI in the recording pipette failed to disrupt mGlu1-LTD in MSNs from the cocaine group (Figure 3A). Notably, the inclusion of the mGlu1 antagonist LY367385 (50 μM) completely abolished the LTD observed in the presence of EVKI (Figure 3A), indicating that this LTD is still mediated by mGlu1 as we observed in cocaine rats in the absence of EVKI (Figure 1). These results indicate that PICK1 is not required for mGlu1-induced CP-AMPAR removal from NAc MSN synapses in cocaine rats.

We also examined if disruption of PICK1 function prevents mGlu1-mediated facilitation of CI-AMPAR-mediated synaptic transmission. To this end, we measured the magnitude of the EPSC amplitude at both negative (EPSC_−70 mV_) and positive (EPSC_+40 mV_) potentials prior to and following bath application of DHPG in the presence of the EVKI. As expected from results in Figure 3A; the presence of EVKI had no effect on DHPG-induced attenuation of the EPSC_−70 mV_ amplitude and accompanying reduction of the RI (Figures 3B–D). However, while DHPG increased the EPSC_+40 mV_ amplitude (reflecting a facilitation of CI-AMPAR transmission), as reported previously (McCutcheon et al., 2011a), it failed to exert such an effect when EVKI was included in the recording electrode (Figures 3B–D). These results demonstrate that mGlu1 activation at NAc MSN synapses recorded on >WD35 from cocaine self-administration induces a PICK1-dependent insertion of CI-AMPARs that normally accompanies—but can be dissociated from—the mGlu1-mediated CP-AMPAR internalization.

## Discussion

During withdrawal from extended-access cocaine self-administration, synaptic transmission in NAc MSNs is altered in a number of significant ways (Wolf, 2016). In addition to accumulation of CP-AMPARs at these synapses (Conrad et al., 2008), we have observed a loss of mGlu5-mediated synaptic depression as well as the emergence of an mGlu1-mediated form of synaptic depression (McCutcheon et al., 2011a). Here, we demonstrated that the latter is a form of LTD (mGlu1-LTD), mediated by the dynamin-dependent endocytosis of CP-AMPARs. Our prior work showed that mGlu1-mediated reduction of CP-AMPAR transmission is accompanied by an enhancement of CI-AMPAR transmission (McCutcheon et al., 2011a). We found that this facilitation requires PICK1, consistent with considerable evidence linking PICK1 to synaptic trafficking of CI-AMPARs (see below). Interestingly, disrupting PICK1 function did not prevent mGlu1-induced CP-AMPAR internalization, indicating that the two halves of the mGlu1-induced swap (CP-AMPAR removal and CI-AMPAR insertion) are not coupled in an obligatory manner in NAc MSNs.

### Significance of mGlu1 LTD for Drug Craving

The mGlu1-mediated attenuation of elevated CP-AMPAR contributions to synaptic transmission is a promising therapeutic target. Thus, we have demonstrated that targeting mGlu1-LTD with systemic or intra-NAc administration of mGlu1 positive allosteric modulators (PAM) removes CP-AMPARs from NAc synapses and through this mechanism inhibits incubated craving for cocaine (Loweth et al., 2014). Systemic mGlu1 PAM administration also inhibits incubated craving for methamphetamine (Scheyer et al., 2016). Others have shown that optogenetic targeting of this LTD in NAc MSNs in a projection-specific manner similarly reduced incubated cocaine craving (Lee et al., 2013; Ma et al., 2014); this approach also reduced cocaine seeking under conditions in which incubation of craving was not demonstrated (Pascoli et al., 2014). Interestingly, one study revealed a contribution of NMDARs to the optogenetic LTD that reduced cocaine craving (Ma et al., 2014), consistent with reports that co-activation of mGlu1 and NMDAR mediate LTD via CP-AMPAR removal in lateral amygdala neurons (Clem and Huganir, 2010), and that activation of extrasynaptic NMDARs, like mGlu1, can induce exchange of CP-AMPARs and CI-AMPARs in cerebellar stellate cells (Sun and June Liu, 2007). However, pharmacological targeting of mGlu1 is more tractable than targeting NMDARs, due to potential excitotoxicity with the latter approach, and we have found that mGlu1 stimulation is sufficient to fully reverse elevation of CP-AMPAR levels in the NAc (McCutcheon et al., 2011a; Loweth et al., 2014; see also Pascoli et al., 2014). Thus, we have focused on elucidating the mechanism by which mGlu1 influences AMPAR transmission in NAc MSNs.

### DHPG Elicits mGlu1 LTD in NAc MSNs That Depends on CP-AMPAR Internalization

We began by comparing the effect of DHPG in NAc MSNs following self-administration of saline or cocaine and a withdrawal period (>35 days) sufficient to induce robust incubation of craving and elevated CP-AMPAR levels in NAc MSNs (Wolf and Tseng, 2012). The synaptic depression induced by DHPG largely recovered after DHPG washout in MSNs recorded from saline control rats, although there was some variability in this response. However, the synaptic depression persisted after DHPG washout (i.e., LTD) in MSNs recorded from rats that had undergone incubation of cocaine craving.

The mechanism by which mGlu1 reduces levels of CP-AMPARs in NAc MSN synapses could involve either internalization or lateral diffusion away from the synapse. Here, we found that mGlu1-LTD is mediated by dynamin-dependent endocytosis of CP-AMPARs. We have previously shown that the CP-AMPARs accumulating during incubation are predominantly homomeric GluA1 receptors (Conrad et al., 2008). Thus, the present results are consistent with the ability of DHPG to produce rapid internalization of GluA1 in cultured NAc MSNs (Mangiavacchi and Wolf, 2004; Loweth et al., 2018). Dynamin-dependent internalization of AMPARs as a mechanism underlying group I mGluR-mediated synaptic depression has been demonstrated in several brain regions (Lüscher and Huber, 2010), and it is well established that this LTD is triggered by mGlu1, in particular when CP-AMPARs are involved (Bellone and Lüscher, 2005, 2006; Mameli et al., 2007, 2009; Kelly et al., 2009; Clem and Huganir, 2010; McCutcheon et al., 2011a).

### mGlu1 Stimulation Elicits PICK1-Dependent CI-AMPAR Insertion Into Synapses

While mGlu1-LTD in the NAc of rats that have undergone incubation of craving is similar to that previously described in the VTA of cocaine exposed rats (Bellone and Lüscher, 2005, 2006; Mameli et al., 2007, 2009; Bellone et al., 2011), our results reveal one difference. In the VTA, disruption of the GluA2-PICK1 interaction prevented mGlu1-LTD; thus, even though CP-AMPARs do not interact with PICK1, their internalization was blocked when PICK1 prevented CI-AMPAR delivery (Mameli et al., 2007). In contrast, while mGlu1-mediated CP-AMPAR removal and CI-AMPAR insertion are normally coupled in NAc MSNs, we found that disruption of PICK1 function prevents CI-AMPAR delivery, but does not prevent CP-AMPAR internalization. Thus, the two events can be dissociated in NAc MSNs. This difference between NAc and VTA may reflect differences in the way cocaine elicits CP-AMPAR accumulation in the two regions; in the NAc, most evidence indicates that CP-AMPAR elevation requires weeks of withdrawal from extended access cocaine self-administration, whereas in the VTA, it occurs within hours of a single cocaine injection (Wolf and Tseng, 2012; Lüscher, 2013; Pignatelli and Bonci, 2015; Wolf, 2016).

## Conclusion

The results of the present study demonstrate the emergence of mGlu1-LTD mediated by the endocytosis of CP-AMPARs in the NAc of rats that have undergone incubation of cocaine craving. Understanding this mechanism is important because of its therapeutic potential. Incubation of craving occurs in rats during withdrawal from many drugs of abuse (cocaine, methamphetamine, nicotine, heroin and alcohol; Pickens et al., 2011), and has been demonstrated in human drug users during withdrawal from cocaine, nicotine, methamphetamine and alcohol (Bedi et al., 2011; Wang et al., 2013; Li et al., 2015; Parvaz et al., 2016). As described above, eliciting mGlu1-LTD (pharmacologically or optogenetically) effectively reduces incubated cocaine or methamphetamine craving by removing CP-AMPARs from synapses (Loweth et al., 2013, 2014). mGlu1 PAMs may also ameliorate cognitive deficits associated with cocaine use (Olive, 2010). Future studies should determine if CP-AMPARs contribute to incubation for other drugs of abuse and whether mGlu1 reverses this incubation. This is timely given the development of improved mGlu1 PAMs for other indications (Garcia-Barrantes et al., 2015).

## Data Availability

All data supporting the conclusions of this manuscript will be made available to any qualified researcher without undue reservation.

## Author Contributions

MW and KT designed the study, wrote the manuscript and prepared the figures. AS and DC performed the experiments and analyzed data under the supervision of KT.

## Conflict of Interest Statement

The authors declare that the research was conducted in the absence of any commercial or financial relationships that could be construed as a potential conflict of interest.
